# A competing-risk nomogram to predict cause-specific death in elderly patients with colorectal cancer after surgery (especially for colon cancer)

**DOI:** 10.1186/s12957-020-1805-3

**Published:** 2020-02-04

**Authors:** Zhengbing Wang, Yawei Wang, Yan Yang, Yi Luo, Jiangtao Liu, Yingjie Xu, Xuan Liu

**Affiliations:** 1 Department of Gastrointestinal Surgery, Affiliated Hospital of Yangzhou University, Yangzhou, 225100 People’s Republic of China; 2 Department of Gastrointestinal Surgery, Northern Jiangsu People’s Hospital, Clinical Medical School, Affiliated Hospital of Yangzhou University, Yangzhou, 225002 People’s Republic of China; 3 Department of General Surgery, Jiangsu Provincial Hospital of Integrated Traditional and Western Medicine, Nanjing, 210046 People’s Republic of China

**Keywords:** Elderly patients, Colorectal cancer, Competing-risk, Nomogram, Prognostic analysis

## Abstract

**Background:**

Clinically, when the diagnosis of colorectal cancer is clear, patients are more concerned about their own prognosis survival. Special population with high risk of accidental death, such as elderly patients, is more likely to die due to causes other than tumors. The main purpose of this study is to construct a prediction model of cause-specific death (CSD) in elderly patients using competing-risk approach, so as to help clinicians to predict the probability of CSD in elderly patients with colorectal cancer.

**Methods:**

The data were extracted from Surveillance, Epidemiology, and End Results (SEER) database to include ≥ 65-year-old patients with colorectal cancer who had undergone surgical treatment from 2010 to 2016. Using competing-risk methodology, the cumulative incidence function (CIF) of CSD was calculated to select the predictors among 13 variables, and the selected variables were subsequently refined and used for the construction of the proportional subdistribution hazard model. The model was presented in the form of nomogram, and the performance of nomogram was bootstrap validated internally and externally using the concordance index (C-index).

**Results:**

Dataset of 19,789 patients who met the inclusion criteria were eventually selected for analysis. The five-year cumulative incidence of CSD was 31.405% (95% confidence interval [CI] 31.402–31.408%). The identified clinically relevant variables in nomogram included marital status, pathological grade, AJCC TNM stage, CEA, perineural invasion, and chemotherapy. The nomogram was shown to have good discrimination after internal validation with a C-index of 0.801 (95% CI 0.795–0.807) as well as external validation with a C-index of 0.759 (95% CI 0.716–0.802). Both the internal and external validation calibration curve indicated good concordance between the predicted and actual outcomes.

**Conclusion:**

Using the large sample database and competing-risk analysis, a postoperative prediction model for elderly patients with colorectal cancer was established with satisfactory accuracy. The individualized estimates of CSD outcome for the elderly patients were realized.

## Background

Colorectal cancer is one of the most common malignancies in Asia and most western countries [1]. It is the third most common cancer in the world with the second highest mortality rate. In 2018, it is estimated that about 1,800,977 people worldwide will develop the disease, of which about 861,663 will die [2]. Various prognostic factors influence the survival outcomes of colorectal cancer patients. For elderly colorectal cancer patients, the probability of death from non-tumor factors, such as cardiovascular and cerebrovascular accidents, severe infections, and underlying diseases, is higher than that of average population, which will hinder the occurrence of death caused by tumor factors. In the case of colon cancer, elderly patients are more likely to develop very rare retroperitoneal colonic perforations and eventually die of severe infection [2]. Therefore, it becomes more difficult for clinicians to predict the prognosis accurately. There is a strong need to develop reliable and discriminative methods to predict the prognosis of elderly patients.

In the era of precision medicine, clinical prediction models, such as the quantitative risk and benefit assessment tool, have been widely used in clinical medical decision-making, patient prognosis management, public health resource allocation, and so on. It is essentially a method of using mathematical formulas to estimate the probability of individual illness or to produce a specific outcome [3–6], which falls under two categories: diagnostic model and prognostic model; the latter has been widely used in the clinical practice to help make more reasonable medical decisions for cancer patients. The prognostic models of clinical outcome can be presented in the form of nomogram, web calculator, scoring system, and so on. Nomogram can be combined with a variety of predictive factors to diagnose or predict the incidence and progression of the disease. The complex statistical model is graphically represented, and the individualized clinical outcome of patients can be quickly estimated without computer software for interpretation/prediction. At present, there have been a number of predictive studies for colorectal cancer. Smith et al. [7] have evaluated 16 diagnostic models for colorectal cancer screening, and Kawai et al. [8] have analyzed the clinical applicability of 28 prognostic models for colorectal cancer.

The concept of competing-risk first appeared in the study of smallpox in the eighteenth century [9] and developed rapidly after Cox [10] put forward the proportional risk model in 1972. Competing-risk refers to the existence of a competitive risk relationship between the former and the latter when there is a known event in the observation queue that may affect the probability of another event or completely hinder its occurrence. The concept of competing-risk is more suitable to the study of elderly patients [11].

In this study, with competing-risk approach, we used SEER database to construct the nomogram of postoperative death probability prediction of elderly patients with colorectal cancer, hoping to help clinicians achieve more personal and accurate prognosis estimation in clinical practice.

## Methods

### Data source and processing

**Fig. 1:**
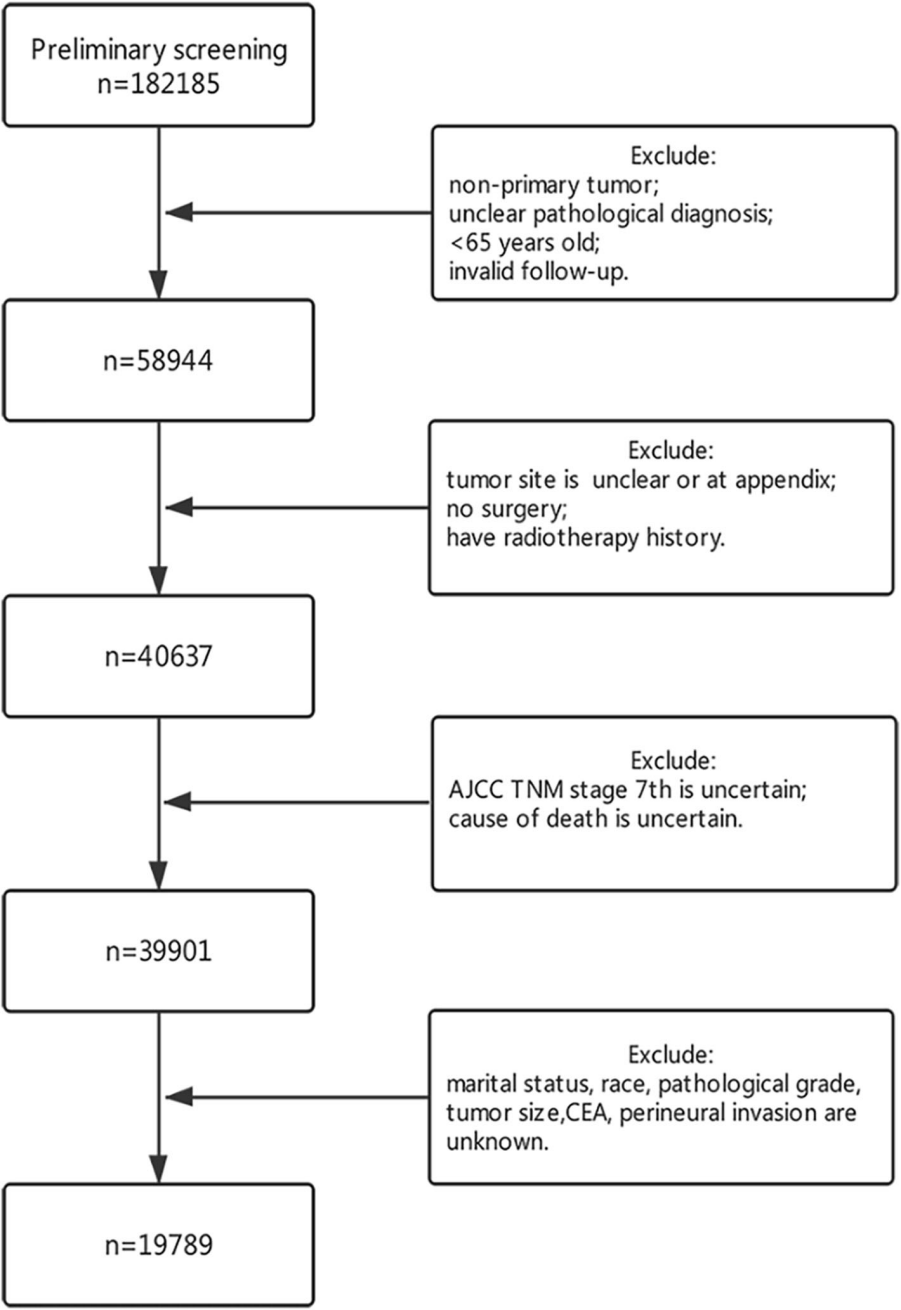
Data screening process

The data were extracted from SEER database (“SEER 18 Regs Custom Data (with additional treatment field), November 2017 Sub (1973 to 2015 varying)” is selected) to include all patients with colorectal cancer who had undergone surgical treatment from 2010 to 2016. Deaths due to cancer were identified by the SEER cause-specific death classification variable. The initial filtering was applied using the following: International Classification of Diseases for Oncology, Third Edition (ICD-O-3), and histology codes: 8020/3, 8032/3, 8070/3, 8140/3, 8201/3, 8213/3, 8480/3, 8490/3, 8510/3, and 8560/3. “Site recode ICD-O-3/WHO 2008” data for filtering tumor location, only including colon and rectum. A total of 182,185 patients were initially obtained, and then were further screened according to the schema shown in Fig. 1. Patients with a follow-up of less than 1 year and the survival outcome alive were considered as invalid follow-up and were excluded. A total of 19,789 patients who met all inclusion criteria were eventually included for analysis.

The external validation data came from 488 patients ≥ 65 years old who received radical resection of colorectal cancer in the Gastrointestinal Surgery Department of Affiliated Northern Jiangsu People’s Hospital to Yangzhou University during the period of August 2012 to August 2016.

### Statistical analysis and construction of the nomogram

Categorical variables in the analysis included marital status, sex, race, tumor site, pathological grade, AJCC TNM stage, CEA, perineural invasion, and chemotherapy. Continuous variables were transformed into categorical variables. Tumor size was stratified by 5 cm cutoff. Marital status was regrouped as married and other status. Race was divided into white and other. Pathological grade was defined as grades I/II and III/IV. CEA was divided into normal and elevated groups. Perineural invasion was divided into negative and positive groups. Due to the limitation of the database, chemotherapy information can only be divided into Yes and No/unknown groups.

CSD and death from other causes are considered to be competitive events. To identify the prognostic parameters that were significantly associated with CSD, the cumulative incidence of each variable could be overestimated if the traditional Kaplan-Meier (K-M) test is used at this time [12]. Under this circumstance, CIF should be calculated for univariate analysis instead of K-M test. CIF calculates the incidence of interest end point events and competitive risk events, and it represents the incidence of interest end point corrected by competitive risk event [13]. We calculated the 5-year CIF of the CSD and plotted the CIF curve. The differences among groups were evaluated by Gray’s test [14]. Median follow-up time was computed by the reverse Kaplan-Meier method. Covariates with statistical differences were selected as candidate predictors and used for the next-step multivariate analysis.

When the competitive risk exists, the use of traditional Cox regression could cause bias; proportional subdistribution hazards regression model is therefore chosen as the appropriate approach [15] for multivariate analysis. Using Cox proportional hazard regression, risk regression solves the problem of competing-risk in risk assessment and can reflect the influence of covariates on cumulative incidence. Nomogram for CSD was formulated based on the results of the multivariate Cox proportional regression analyses.

### Validation and calibration of the nomogram

The performance of the prediction model was validated internally and externally by bootstrap method. C-index was calculated to access discrimination [16], and the calibration was evaluated with the calibration curve [17].

### Tools and software

Data extraction is based on SEER*Stat version 8.3.5. Median follow-up time calculated using SPSS version 24.0. In the R software 3.5.0 version, cuminc () function in cmprsk software package was used for univariate analysis, and crr () function for multivariate analysis. Nomogram was drawn by referring to the step-by-step method provided by Zhang et al. [18]. Nomogram was plotted with crprep () function in mstate package, cph () function, and nomogram () function in rms package. Finally, calibrate () function in rms package and rcorr.cens () function in Hmisc package were used to evaluate the performance of the model. All *P* values were obtained by two-sided statistical testing.

## Results

**Fig. 2:**
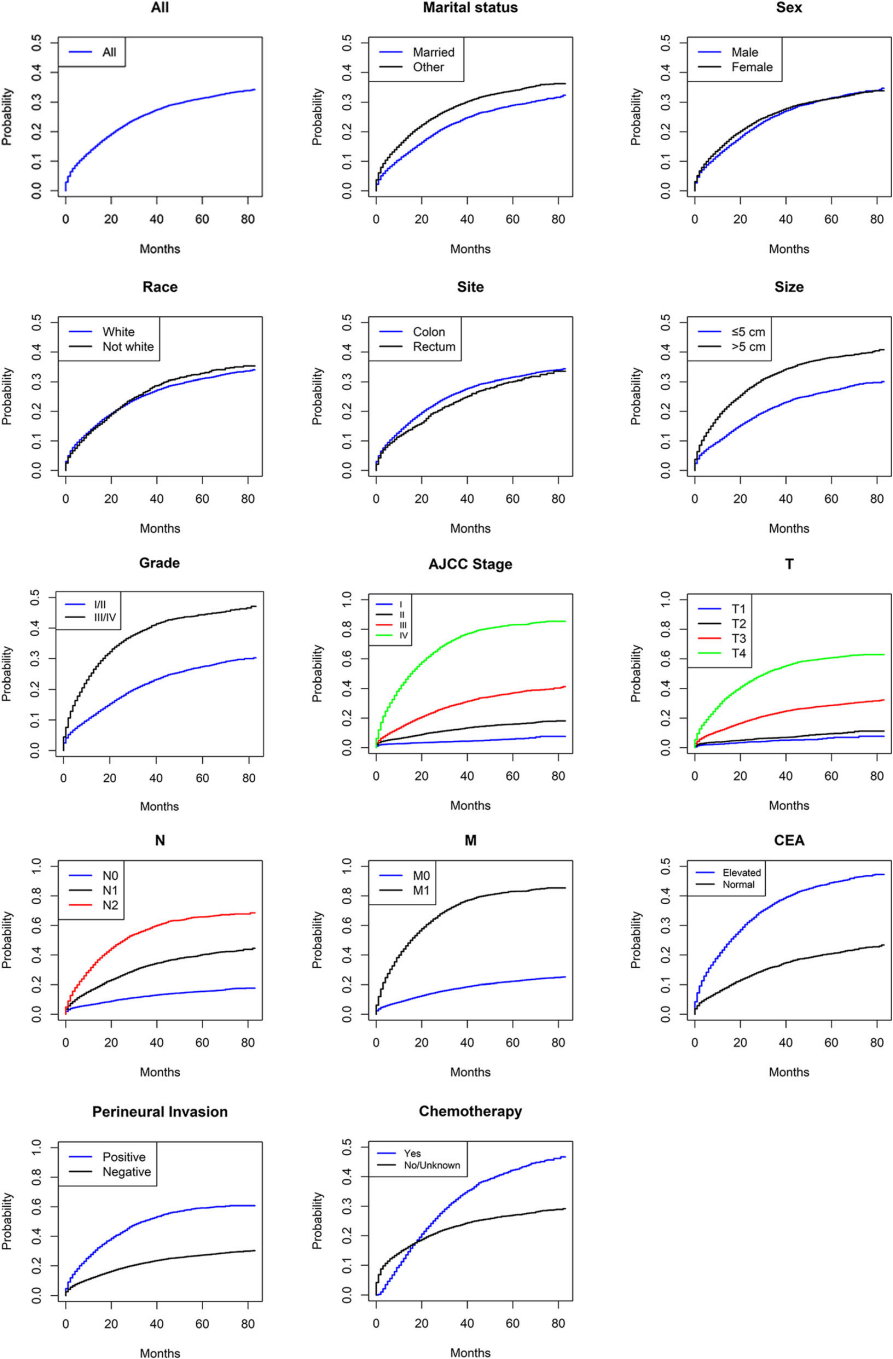
CIF curve of death according to patient characteristics

The median follow-up period was 46 months. Table 1 shows the demographic and tumor characteristics for the cohort of 19,789 patients with colorectal cancer, as well as univariate analysis of the 5-year cumulative incidences of CSD. The CIF curve is shown in Fig. 2.

**Table 1:** The five-year cumulative incidences of death among elderly patients with colorectal cancer after surgery

Characteristics	*N* (%)	Cancer-specific death		
		*N* (%)	5-year (%) (95% CI)	*P* value
Total	19,789	5417	31.405 (31.402–31.408)	
Marital status				< 0.001
Married	10,209 (51.589)	2527 (46.649)	29.014 (29.009–29.02)	
Other	9580 (48.411)	2890 (53.351)	33.948 (33.942–33.954)	
Sex				0.152
Male	9229 (46.637)	2484 (45.856)	31.543 (31.537–31.55)	
Female	10,560 (53.363)	2933 (54.144)	31.307 (31.302–31.313)	
Race				0.235
White	16,030 (81.005)	4361 (80.506)	31.071 (31.067–31.074)	
Not white	3759 (18.995)	1056 (19.494)	32.868 (32.852–32.884)	
Tumor site				0.033
Colon	17,774 (89.818)	4901 (90.474)	31.548 (31.544–31.551)	
Rectum	2015 (10.182)	516 (9.526)	30.108 (30.08–30.137)	
Size, cm				< 0.001
≤ 5	12,050 (60.892)	2804 (51.763)	27.08 (27.076–27.085)	
> 5	7739 (39.108)	2613 (48.237)	38.183 (38.175–38.191)	
Pathological grade				< 0.001
I/II	15,138 (76.497)	3526 (65.091)	27.413 (27.41–27.417)	
III/IV	4651 (23.503)	1891 (34.909)	44.374 (44.361–44.387)	
AJCC stage				< 0.001
I	3130 (15.817)	159 (2.935)	5.828 (5.823–5.833)	
II	7175 (36.258)	979 (18.073)	15.923 (15.918–15.928)	
III	6529 (32.993)	2053 (37.899)	37.113 (37.103–37.123)	
IV	2955 (14.933)	2226 (41.093)	83.002 (82.988–83.016)	
AJCC T stage				< 0.001
T1	884 (4.467)	47 (0.868)	6.5 (6.48–6.521)	
T2	2875 (14.528)	228 (4.209)	9.475 (9.467–9.483)	
T3	12,018 (60.731)	2981 (55.03)	28.747 (28.743–28.752)	
T4	4012 (20.274)	2161 (39.893)	60.796 (60.78–60.812)	
AJCC N stage				< 0.001
N0	10,780 (54.475)	1444 (26.657)	15.488 (15.485–15.491)	
N1	5425 (27.414)	1864 (34.41)	40.294 (40.281–40.306)	
N2	3584 (18.111)	2109 (38.933)	65.79 (65.773–65.807)	
AJCC M stage				< 0.001
M0	16,834 (85.067)	3191 (58.907)	22.228 (22.225–22.231)	
M1	2955 (14.933)	2226 (41.093)	83.002 (82.988–83.016)	
CEA				< 0.001
Elevated	9001 (45.485)	3526 (65.091)	44.51 (44.502–44.517)	
Normal	10,788 (54.515)	1891 (34.909)	20.477 (20.473–20.481)	
Perineural invasion				< 0.001
Positive	2621 (13.245)	1345 (24.829)	59.2 (59.174–59.227)	
Negative	17,168 (86.755)	4072 (75.171)	27.284 (27.281–27.288)	
Chemotherapy				< 0.001
Yes	5816 (29.390)	2019 (37.272)	42.267 (42.255–42.28)	
No/unknown	13,973 (70.61)	3398 (62.728)	26.959 (26.955–26.962)

The mean age of the 19,789 patients was 76.451 ± 7.698 years, and 35.621% were over 80. The majority of the study population is female (53.363%), white (81.005%), and marital status “married” (51.589%). Most tumors occurred in the colon (89.818%). Tumor size less than 5 cm (60.892%) and pathological grade I/II (76.497%) accounted for the majority. AJCC TNM stage I, stage II, stage III, and stage IV accounted for 15.817%, 36.258%, 32.993%, and 14.933%, respectively. The AJCC T stage distribution was T1 (4.467%), T2 (14.528%), T3 (60.731%), and T4 (20.274%), respectively. Lymph node metastasis occurred in 45.525% of the patients, and 14.933% of the patients had distant metastasis. Patients with elevated CEA and positive perineural invasion accounted for 45.485% and 13.245%, respectively. 29.390% of the patients were clearly treated with chemotherapy.

A total of 7918 deaths were included, including 5417 CSDs. The 5-year cumulative incidence of the CSD was 31.405% (95% CI 31.402–31.408%). Patients who were married, with tumors less than 5 cm, lower pathological grades, earlier TNM stages, normal CEA, and negative perineural invasion had a lower 5-year cumulative incidence of CSD with statistically significant difference (*P* < 0.001). The 5-year cumulative incidence of CSD was different in patients with different treatment regimens after operation. Mortality rate was higher in patients undergoing chemotherapy (*P* < 0.001). This may be due to the fact that patients receiving chemotherapy tend to be in late stage as well as the vulnerability of elderly patients to chemotherapy. There was no significant difference in the 5-year cumulative incidence of CSD in sex and race (*P* = 0.152, 0.235, respectively), and the CIF curves among the two groups were similar. The covariates of sex and race were therefore excluded, and the remaining indicators were incorporated into multivariate analysis. In addition, although univariate analysis of tumor site and AJCC T stage showed a statistically significant difference in the 5-year cumulative incidence of CSD, the difference in the CIF curve is not obvious, and predictive power of these factors will be further examined in the subsequent multivariate analysis.

Marital status, tumor site, tumor size, pathological grade, TNM stage, CEA, perineural invasion, and chemotherapy were included in multivariate analysis, and proportional subdistribution hazard regression was used to filter covariates with statistical differences. In the first round of multivariate analysis, the covariates of tumor site had no significant difference (*P* = 0.900). No significant difference was observed between T2 and T1 (*P* = 0.150), and the subdistribution hazard ratio (sdHR) of stage T2 vs. T1 was 1.257 (95% CI 0.944–1.570). We also noticed that the scores corresponding to T1 and T2 in nomogram were very close. To simplify the model, T1 and T2 were combined into one group. Eventually, T staging was divided into three classification variables (T1/2, T3, T4); tumor site was excluded and all other covariables remained unchanged.

The sdHRs of CSD in elderly colorectal cancer based on the competing-risk model are shown in Table 2.

**Table 2:** Proportional subdistribution hazard models of probabilities of cancer-specific death for elderly patients with colorectal cancer after surgery

Characteristics	sdHR	95% CI	*P* value
Marital status			
Married	1		
Other	1.200	1.145–1.255	< 0.001
Size, cm			
≤ 5	1		
> 5	1.09	1.035–1.146	0.002
Pathological grade			
I/II	1		
III/IV	1.322	1.262–1.382	< 0.001
AJCC T stage			
T1/T2	1		
T3	2.162	2.034–2.289	< 0.001
T4	3.432	3.293–3.570	< 0.001
AJCC N stage			
N0	1		
N1	2.289	2.211–2.367	< 0.001
N2	3.297	3.211–3.383	< 0.001
AJCC M stage			
M0	1		
M1	3.554	3.484–3.623	< 0.001
CEA			
Normal	1		
Elevated	1.447	1.388–1.507	< 0.001
Perineural invasion			
Negative	1		
Positive	1.24	1.172–1.308	< 0.001
Chemotherapy			
Yes	1		
No/unknown	1.994	1.931–2.057	< 0.001

Multivariate results showed that the covariates included had strong predictive effect on CSD. Comparing marital status of “other” with “married” patients, the sdHR of the former was 1.200 (95% CI 1.145–1.255). The result showed that married patients have a better prognosis. Higher pathological grades and larger tumor size were associated with an increased probability of CSD. Compared with I/II grade, the sdHR of III/IV grade was 1.322 (95% CI 1.262–1.382). Compared with tumor size ≤ 5 cm, the sdHR of tumor size > 5cm was 1.090 (95% CI 1.035–1.146). In TNM staging, the cause-specific mortality in T3 and T4 was higher than that in T1/T2, with sdHR of 2.162 (95% CI 2.034–2.289) and 3.432 (95% CI 3.293–3.570), respectively. Lymph node positivity and distant metastasis were associated with an increased probability of CSD. Compared with N0, the sdHR of N1 and N2 was 2.289 (95% CI 2.211–2.367) and 2.297 (95% CI 3.211–3.383), respectively. The sdHR of M1 was 3.554 (95% CI 3.484–3.623) compared with M0. Elevated CEA and positive perineural invasion were indicators for poor prognosis, and when compared with normal CEA and negative perineural invasion, sdHR was 1.447 (95% CI 1.388–1.507) and 1.240 (95% CI 1.172–1.308), respectively. In multivariate analysis, patients with chemotherapy were found to have a better prognosis, and sdHR of 1.994 (95% CI 1.931–057) was observed in patients with No/unknown chemotherapy compared with those with chemotherapy. This result shall not be simply considered as contradictory to the outcome of univariate analysis, but rather can be interpreted as the patient benefiting from chemotherapy.

**Fig. 3:**
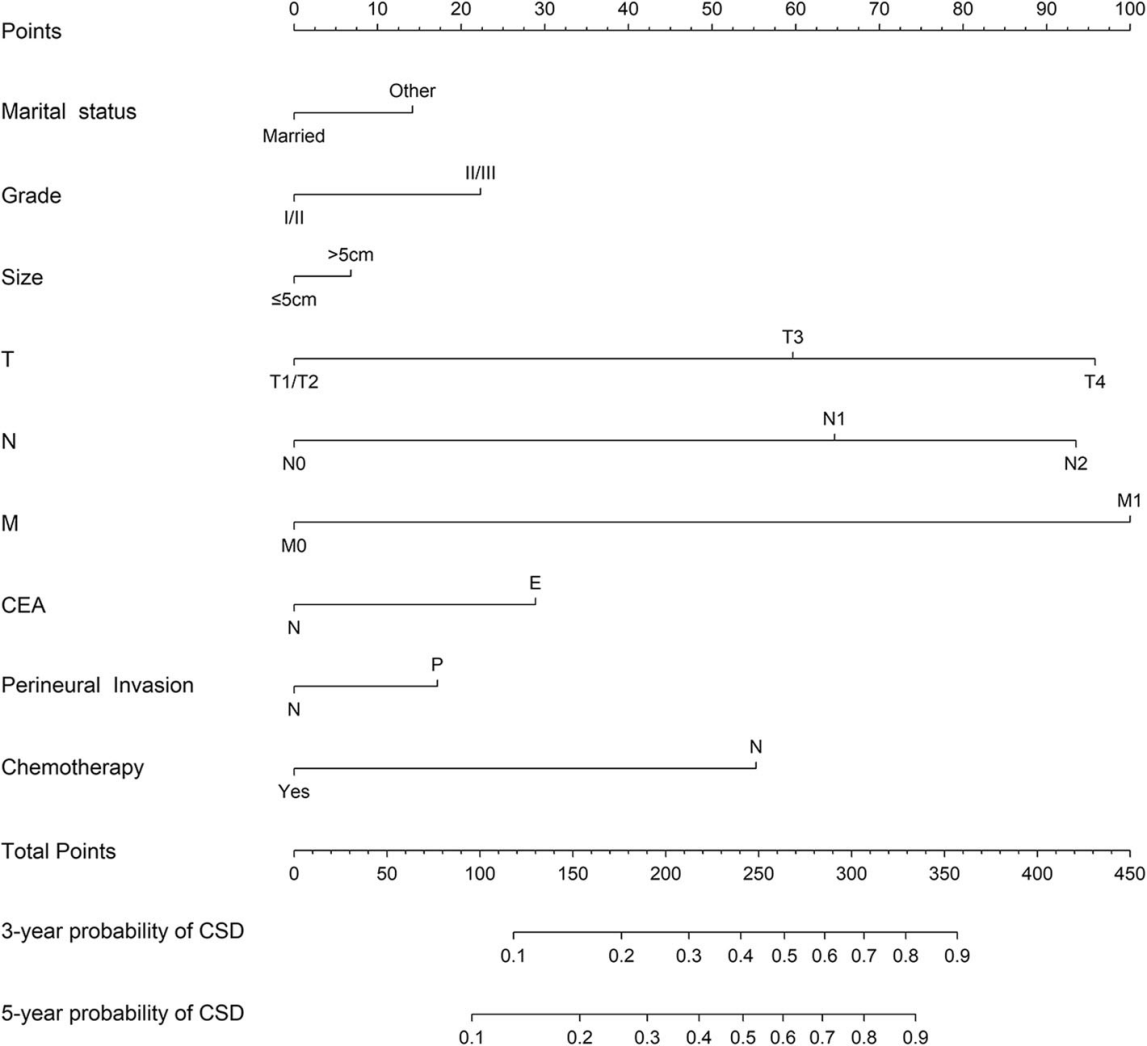
Nomogram for CSD in elderly patients with colorectal cancer after surgery

All above covariates demonstrated statistical difference in the multivariate analysis, so they were included in the construction of the nomogram. The nomogram in Fig. 3 shows the predicted probability of CSD in elderly colon cancer after surgery based on Fine and Gray’s regression. The nomogram was characterized by 1 scale corresponding to each variable, a score scale, a total score scale, and a probability scale. The use of the nomogram is simple and involves 3 steps. First, on the scale for each variable, make a vertical line from each positioning point to the upper point line to obtain the score corresponding to each variable. Second, add up all the scores obtained in the previous step to get the total score. Finally, the probability of CSD of 3-year and 5-year corresponding to the total score of the subject is read on the probability scale.

**Fig. 4:**
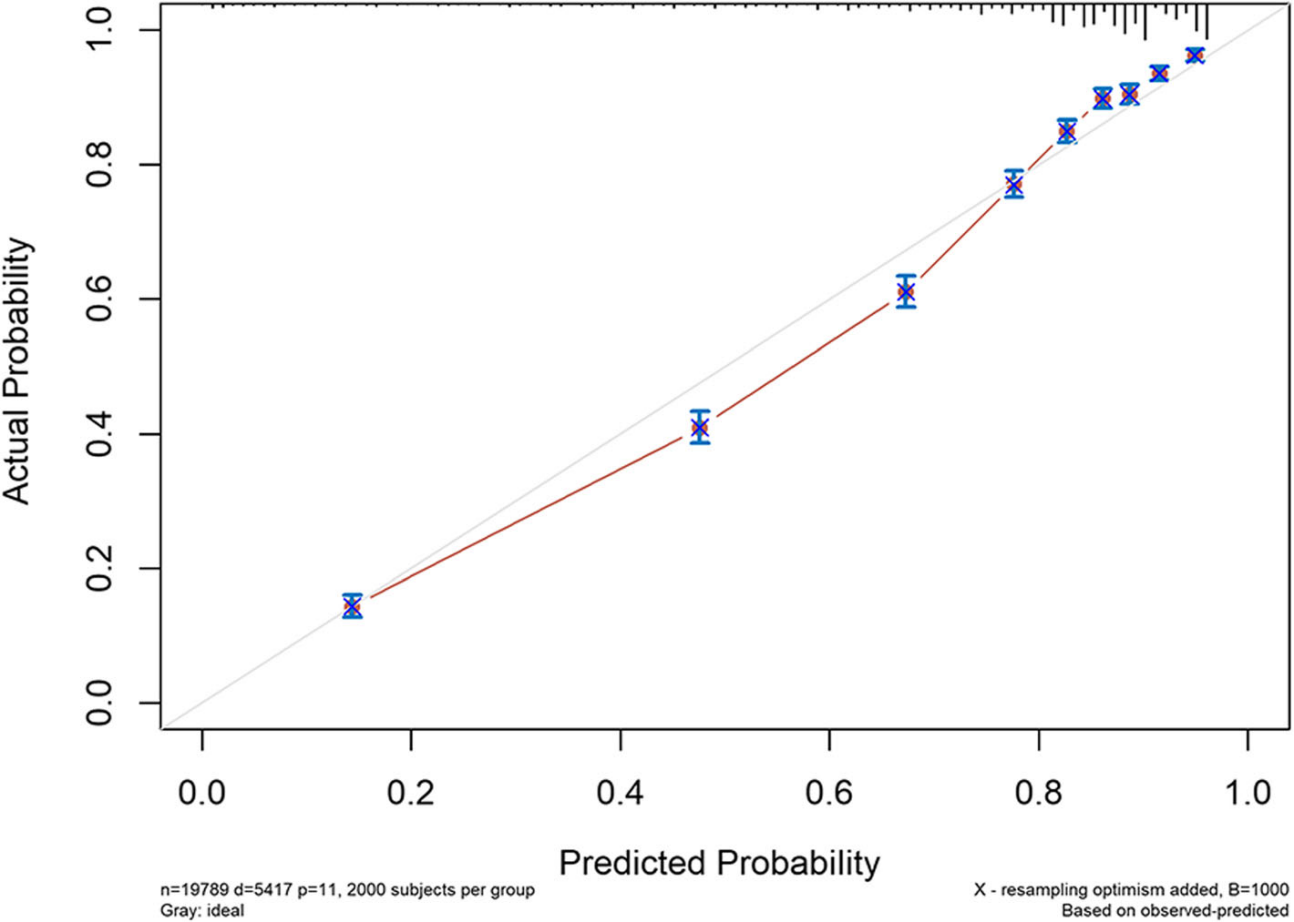
Internal validation calibration curve for the prediction of 3-year CSD

**Fig. 5.**
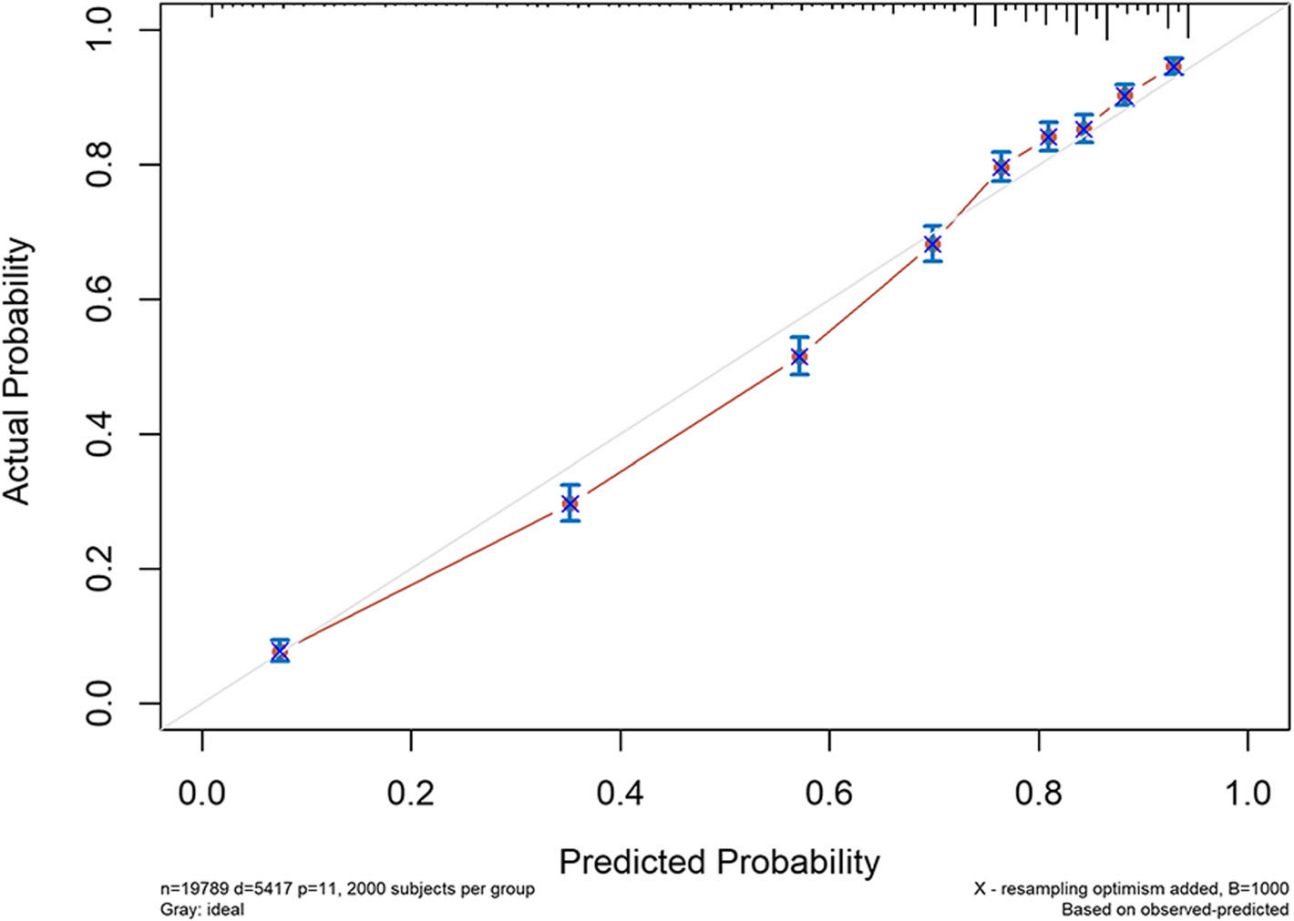
Internal validation calibration curve for the prediction of 5-year CSD

The model was found to have adequate discrimination in internal validation with a C-index of 0.801 (95% CI 0.795–0.807), and the calibration curves of 3-year and 5-year probability of CSD are shown in Figs. 4 and 5, respectively. The predicted mortality had a good correlation between the prediction by nomogram and actual observation.

**Fig. 6:**
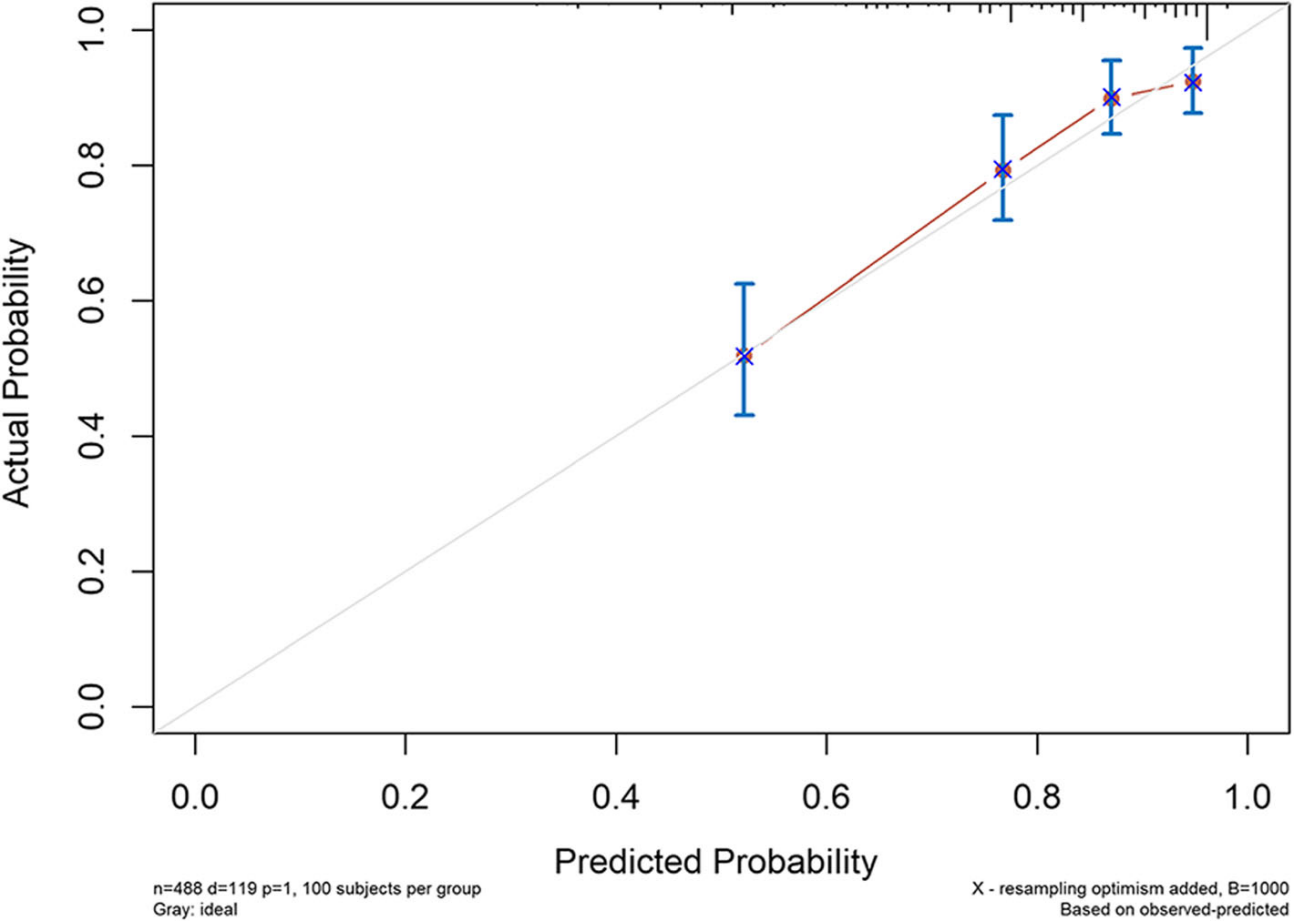
External validation calibration curve for the prediction of 3-year CSD

**Fig. 7:**
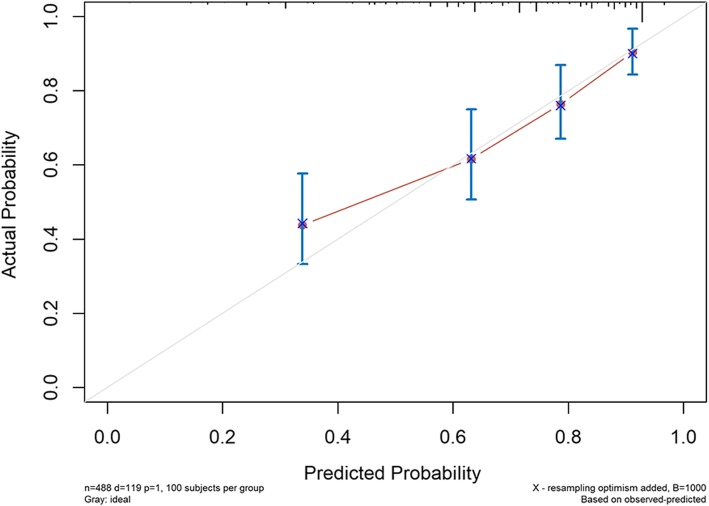
External validation calibration curve for the prediction of 5-year CSD

The independent cohort of 488 colorectal cancer patients aged ≥ 65 years treated at the Gastrointestinal Surgery Department of Affiliated Northern Jiangsu People’s Hospital to Yangzhou University during the period of August 2012 to August 2016 were used for external validation, and their characteristics are summarized in Table 3. As of August 2019, of the 488 follow-up patients, 119 had died of tumors and 352 remain alive. The median follow-up period was 47 months. C-index for external validation was 0.759 (95% CI 0.716–0.802). The calibration curves for 3-year and 5-year probability of CSD are shown in Figs. 6 and 7. The discrimination and calibration were good both in internal and external validation, indicating that the prediction model constructed in this study would have good application value.

**Table 3:** Characteristics of external validation data

External validation set (*n* = 488)		
Characteristics	Number of patients	%
Marital status		
Married	433	88.730
Other	55	11.270
Size, cm		
≤ 5	330	67.623
> 5	158	32.377
Pathological grade		
I/II	413	84.631
III/IV	75	15.369
AJCC T stage		
T1/T2	112	22.951
T3	80	16.393
T4	296	60.656
AJCC N stage		
N0	298	61.066
N1	145	29.713
N2	45	9.221
AJCC M stage		
M0	484	99.180
M1	4	0.820
CEA		
Elevated	194	39.754
Normal	294	60.246
Perineural invasion		
Positive	42	8.607
Negative	446	91.393
Chemotherapy		
Yes	182	37.295
No/unknown	306	62.705

## Discussion

Analyzing the clinical follow-up data, we often encounter the situation that the end point event has not been observed when the study ends for various reasons. When this happens, the occurrence time of the failure event can only be determined to be after the recorded time, which is called right censoring. In cancer patients, the existence of right censoring is caused by a variety of reasons, such as loss of visit and no death. The occurrence of these events does not prevent the survival or death of the patient. However, when a patient dies from causes such as cardiovascular and cerebrovascular accidents, serious infections, and car accidents, during follow-up, the occurrence of CSDs is prevented. If we continue to use this kind of right censored data with traditional survival analysis for regression analysis, there will be bias, and often lead to overestimation of the probability of tumor-related deaths. Unfortunately, these is the issue which frequently occurs when making prognostic prediction for elderly patients as old population possesses a high frequency of frailty and comorbidities, exhibiting increased mortality from other causes among those with cancer.

If we adhere to the traditional survival analysis method, we have to eliminate the patients with non-colorectal cancer-specific death, which on the one hand, it will lead to the reduction of sample size, and the survival data of the excluded data will not be available. On the other hand, it can lead to selective bias, and those who are older and have a lot of underlying diseases are often eliminated, making the included population unrepresentative.

At this time, the use of competing-risk concept can be a good solution to this problem. In the case of competing-risk, single univariate analysis can be carried out by calculating the CIF of concern events and competitive events. CIF assumes that there is one and only one occurrence of each event. The sum of CIFs of each category is equal to the composite event CIF. The obtained event of interest rate is corrected by competing-risk.

In multivariate analysis, the two most commonly used methods are cause-specific hazard function and proportional subdistribution hazard function. The biggest difference between the two lies in the definition of “risk set”; the latter integrates competitive outcome into the definition of risk set and is only interested in the absolute incidence of the end point of interest, which will help to establish a direct relationship between covariates and CIF. Proportional subdistribution hazard function makes the covariant effect a better and more intuitive explanation and is suitable for the establishment of clinical prediction model and risk score [19]. The cause-specific hazard function, on the other hand, is more suitable for etiological study [20]. In addition, the method proposed by Klein and Andersen can also be used for multivariate analysis under competing-risk, and their results were similar to those of proportional subdistribution hazard function [21].

The previous clinical scoring system uses individual risk factors. The advantage is that it is simple and convenient. However, due to the fact that each risk factor is weighted equally and the information loss can happen in the process of variable data conversion, the accuracy of this scoring system is not yet optimal [22]. The nomogram approach can help avoid these disadvantages. As a visual tool, the nomogram can provide more accurate and quantitative prediction results for specific patients. It has rich clinical significance in the occurrence, outcome, prognosis, and recurrence of the disease [23]. At the same time, because the variables can be obtained in the clinical setting, the prediction tool is practical and convenient.

In terms of predicative factors, marital status is often found to be valuable in many tumor-related survival analysis studies. Similar to the reported findings [24], we also concluded that married patients have a better prognosis than those who are not. Although our nomogram shows a relative low weight of tumor size in the influence of prognosis, the predictive trend is consistent with reported studies that tumor size is negatively correlated with survival rate, which reflects the invasiveness of tumor to a certain extent [25, 26]. Pathological grade and TMN stage are known to affect the prognosis of the patients, such effect is demonstrated by heavy weights of these factors in the nomogram, although some studies has suggested that the existing N staging system may have limitation, and emphasis should be placed on the value of lymph node ratio in prognosis [27, 28]. Lymph node ratio, N stage, the number of lymph nodes detected, tumor deposits, and other lymphatic related indicators are still the focus of discussion [29–31]. Preoperative CEA has been widely recognized as an independent prognostic factor for colorectal cancer, which can effectively predict the prognosis of colorectal cancer [32]. This factor indeed shows a high contribution coefficient in our nomogram. Perineural invasion is a possible pathway for metastatic diffusion of tumors, which can lead to poor prognosis of tumors [33, 34]. The prognostic value of this variable is also reflected in our model. One of the significantly weighted variables in the prediction model is chemotherapy status. Despite the fact that SEER database does not capture enough information about non-chemotherapy, it is clear from the model that patients undergoing chemotherapy has better prognosis [35]. However, there is still controversy as to under what circumstances chemotherapy is needed in elder patients to maximize the benefits.

Among the predicative factors that are not included in our model, lymphovascular invasion (LVI) is worth mentioning. Although LVI is a known risk factor on prognosis in patients with colorectal cancer, such information is not captured in the SEER database. On the other hand, colorectal cancer circumferential resection margin information is available in the SEER database; this variable is not considered as an appropriate prognostic factor [33], and our model did not include this indicator. Socioeconomic factors such as “insurance recode” were not selected either as such factor is influenced by insurance policy in different area, may result in bias and affect the applicability of the predictive model.

This study is mainly concerned with the prognosis of elderly patients with colorectal cancer after surgery. Radiotherapy may have a greater impact on the prognosis [36–38]. In order to avoid this effect, we removed all patients who received radiotherapy at any time. When radiotherapy was an excluding criterion for filtering study population, more patients with rectal cancer were removed, since radiotherapy is more often used to treat patients with rectal cancer than for colon cancer, which may cause the patient population of rectal/colon cancer patients (90%/10%) in the study cohort to be different from the anatomical distribution of colorectal cancer in general population. So the model we built may be more suitable for patients with colon cancer.

Prediction model plays an important role in medical decision-making [39]. The use of prognostic and decision aids in cancer treatment, e.g., nomograms, has grown rapidly in the last decade. There are many studies on nomograms; some affirmed the importance of nomograms [40], while some raised doubts [41]. The overall message is that nomogram should be applied to cohort with similar demographic and disease outcomes, so that the prediction bias caused by patient population can be reduced [42]. In addition, a robust nomogram requires rigorous validation and consistent verification, and giving pros/cons equal attention. Finally, nomograms can be used in conjunction with comprehensive geriatric assessment for older cancer patient to help select the most appropriate treatment. In clinical work, we try to use this nomogram after surgery. Although the accuracy of the nomogram needs further verification, the patient’s medical compliance has improved significantly. It can help us achieve more effective follow-up of patients, which is of great clinical significance.

The prediction model constructed presented in this study has the following advantages as a clinical tool. Firstly, individualized risk predictions for specific events in cancer patients are beneficial for patient counseling and clinical decision-making. The nomogram developed in this study is convenient for guiding clinicians in the exercise of clinical follow-up of patients and the formulation of treatment plans. For example, patients with a high probability of CSD prediction can be reminded to carry out the necessary follow-up at a special time, clarify the postoperative status, guide treatment, and evaluate the results of intervention therapy. Such practice will help to establish a positive and effective medical relationship. Secondly, in today’s big data era, the change of tumor information can be reflected by big data’s continuous update. We can constantly optimize our model according to the continuous updating of the SEER database in the future. And in this process, we can find a certain trend of tumor development and provide reliable data support for a wider range of clinical work. Thirdly, the construction method of the model is suitable for practice in different regions, which helps to build a more regionally representative prediction model.

There are also some limitations in our research. First of all, the SEER database itself as a high-quality large-scale population-based cancer registry [43], and the relevant information is still not perfect, such as patients’ BMI, eating habits, chemotherapy information, and underlying diseases. The following extrinsic factors, such as the difference in the operation itself and the occurrence of postoperative complications, are related to the doctor’s skill level and the hospital’s medical facilities, and are difficult to be reflected in the database. Data before 2010 have more missing information, and we cannot include them for analysis. Second, in terms of statistical analysis, due to the large amount of data, when the *P* value is around 0.05, estimate should be interpreted with caution considering its statistical significance [43]. The short follow-up time of some censored data will also have a statistical impact on the prediction model. Third, the predicted values obtained from this study are not absolutely accurate and shall only be used as a reference to assist clinical decision-making. Finally, this model has more advantages in colon cancer.

## Conclusions

With the help of competing-risk model, we have successfully constructed a nomogram for predicting the postoperative survival of elderly patients with colorectal cancer. The internal and external validation of the line diagram showed the accuracy of the model, which has certain guiding significance for clinical work. The goal is to help clinicians achieve accurate prediction of the prognosis of elderly cancer patients.

## Data Availability

The data for constructing model were obtained from the SEER database. The data for external validation were obtained from the Department of Gastrointestinal Surgery, Northern Jiangsu People’s Hospital, Affiliated Hospital of Yangzhou University

## Abbreviations

CIF: Cumulative incidence function
C-index: Concordance index
CSD: Cause-specific death
ICD-O-3: International Classification of Diseases for Oncology, Third Edition
K-M: Kaplan-Meier
sdHRs: Subdistribution hazard ratios
SEER: Surveillance, Epidemiology, and End Results
